# Supplementary material for: Minimal immune cell subset differences in a cohort of close contacts of tuberculosis index cases
Source: Tuberculosis (Edinb). Author manuscript; Available in PMC 2026 Jul 16. (PMC13373719; doi:10.1016/j.tube.2025.102707)
Supplement: 1 [file NIHMS2186814-supplement-1.docx]

**Gating strategy**

**Supplementary Figure 1**


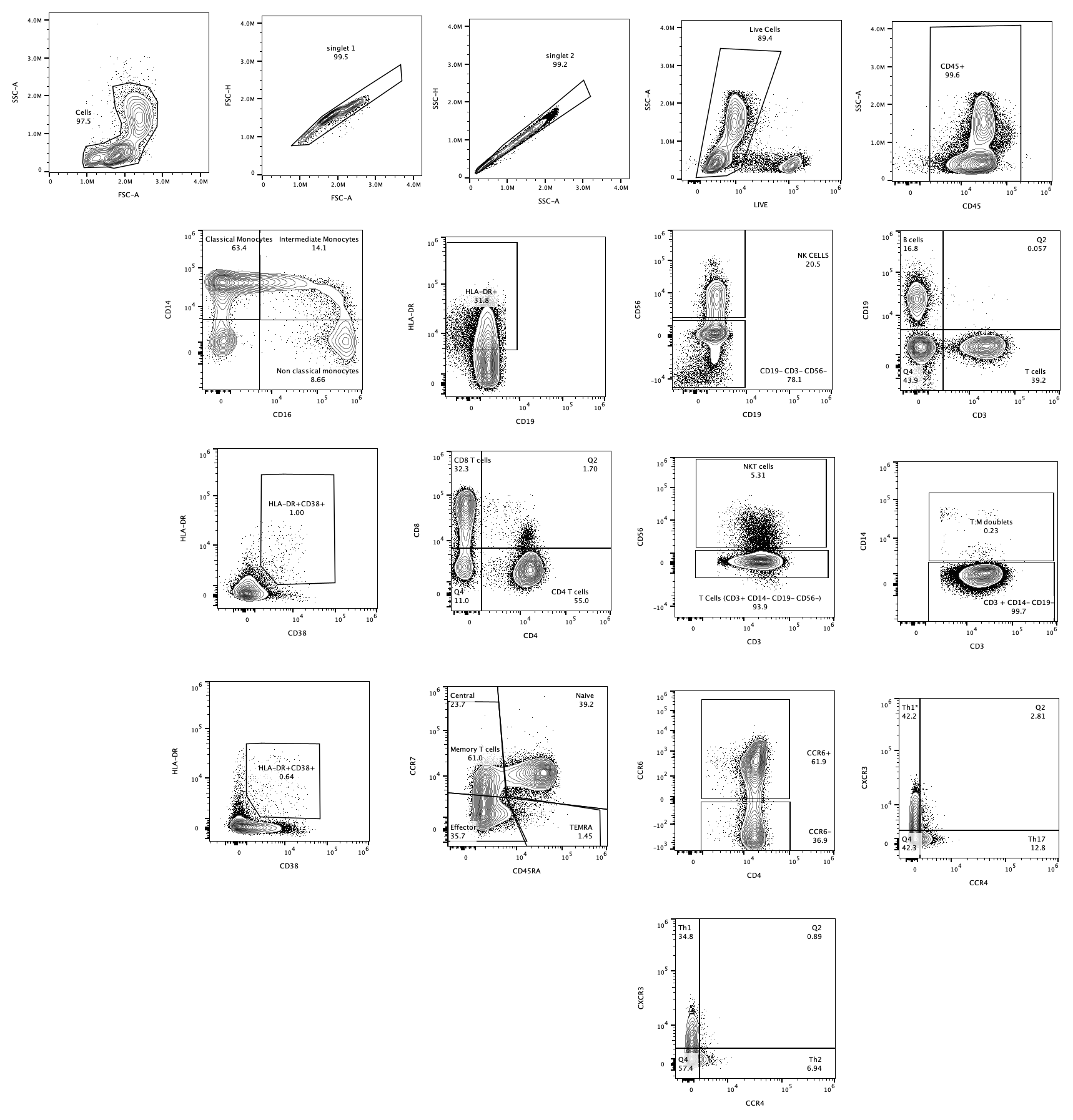


(A)

**Figure S1:** Representative flow cytometry plots showing each cell population assessed in the study. Gating was done on total cells, followed by sequential exclusion of doublets using FSC-A vs. FSC-H (Singlet 1) and SSC-A vs. SSC-H (Singlet 2). Live cells were identified using Live/Dead stain vs. SSC-A and gated on CD45 to define total leukocytes. Within CD45⁺ cells, CD3 vs. CD19 gating was used to identify T cells (CD3⁺) and B cells (CD19⁺), while CD3⁻CD19⁻ cells were further gated on CD56 to define NK cells (CD56⁺) and non-NK myeloid cells (CD56⁻). The CD3⁻CD19⁻CD56⁻ population was then gated on HLA-DR, and HLA-DR⁺ cells were further characterized based on CD14 and CD16 to define monocyte subsets. Within the CD3⁺ population, cells were also gated on CD14 to identify T: monocyte (T:M) doublets (CD3⁺CD14⁺), and the CD3⁺CD14⁻ population was further gated on CD56 to distinguish NKT cells (CD3⁺CD56⁺) from conventional T cells (CD3⁺CD56⁻CD14⁻). T cells were then gated on CD4 and CD8 to define CD4⁺ and CD8⁺ subsets, with activation status assessed by HLA-DR and CD38 co-expression. CD4⁺ T cells were further characterized using CD45RA and CCR7 into naïve (CCR7⁺CD45RA⁺), central memory (CCR7⁺CD45RA⁻), effector memory (CCR7⁻CD45RA⁻), and TEMRA (CCR7⁻CD45RA⁺) subsets. Non-naïve CD4⁺ memory cells were subsequently gated on CCR6, CXCR3, and CCR4 to define Th1 (CCR6⁻CXCR3⁺CCR4⁻), Th2 (CCR6⁻CXCR3⁻CCR4⁺), Th17 (CCR6⁺CXCR3⁻CCR4⁺), and Th1* (CCR6⁺CXCR3⁺CCR4⁻) populations.

**Supplementary figure 2**


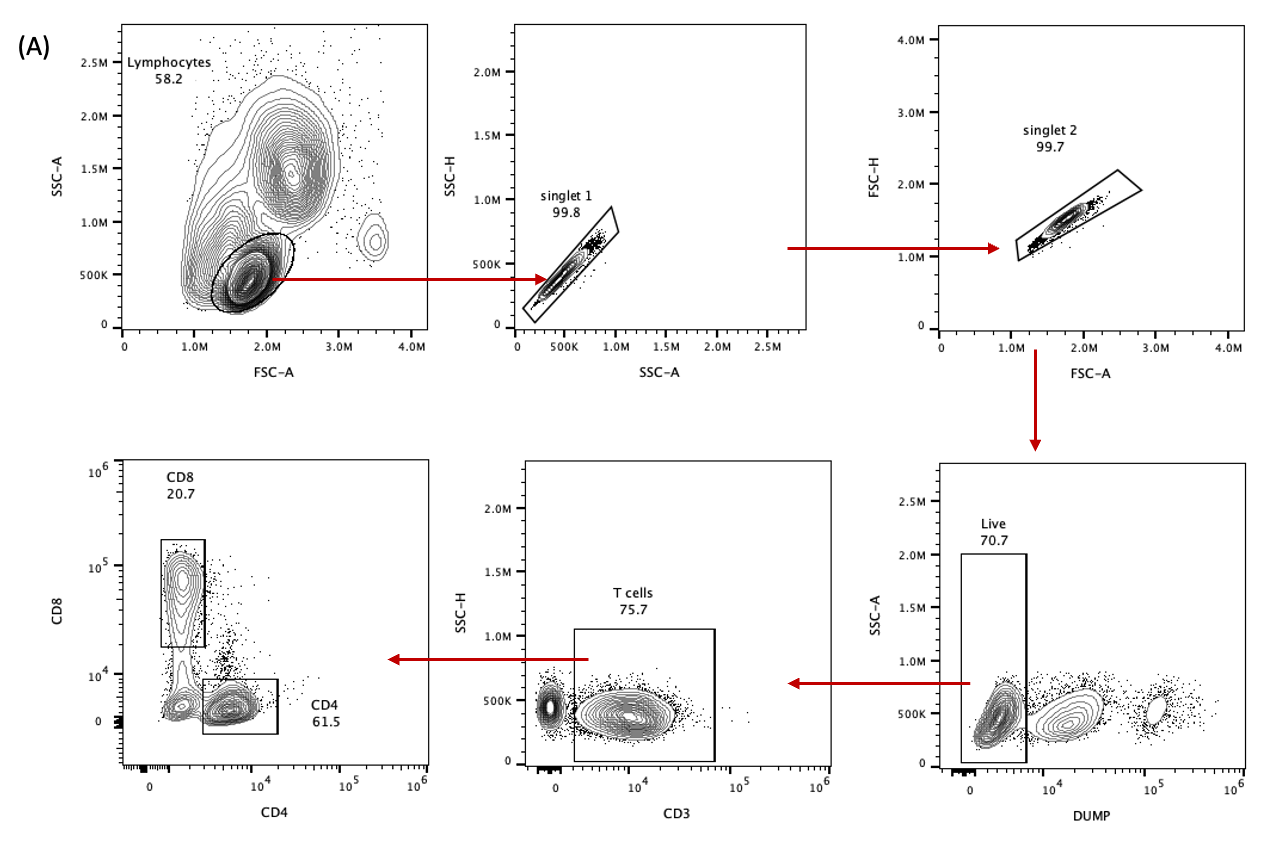


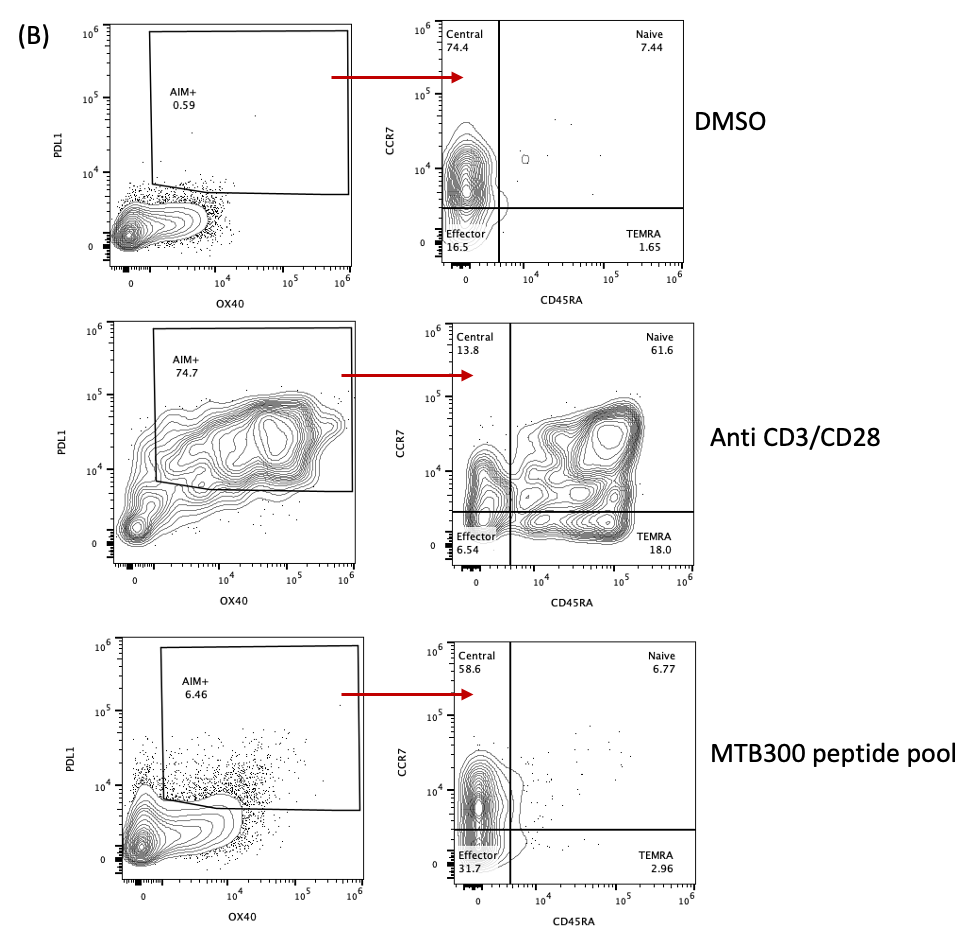


**Figure S2:** Representative flow cytometry plots showing gating strategy for T cells. (A) Gating was done on lymphocytes, followed by sequential exclusion of doublets using SSC-A vs. SSC-H (Singlet 1) and FSC-A vs. FSC-H (Singlet 2). Dead cells and non-T cells were excluded using a DUMP channel (including Live/Dead stain, CD14⁺ monocytes, and CD19⁺ B cells). DUMP-negative cells were gated on CD3⁺ T cells, which were subsequently divided into CD4⁺ and CD8⁺ subsets.

(B) CD4⁺ T cells were analyzed for activation-induced marker (AIM) expression based on dual positivity for OX40 and PD-L1. AIM⁺ CD4⁺ T cells (OX40⁺PD-L1⁺) were then gated on CD45RA and CCR7 to define memory subsets: central memory (CCR7⁺CD45RA⁻), effector memory (CCR7⁻CD45RA⁻), naïve (CCR7⁺CD45RA⁺), and TEMRA (CCR7⁻CD45RA⁺). PBMCs were stimulated with DMSO (negative control), anti-CD3/CD28 (positive control), or MTB300 peptide pool (test condition).
