# Supplementary material for: Minimal immune cell subset differences in a cohort of close contacts of tuberculosis index cases
Source: Tuberculosis (Edinb). Author manuscript; Available in PMC 2026 Jul 16. (PMC13373719; doi:10.1016/j.tube.2025.102707)
Supplement: 2 [file NIHMS2186814-supplement-2.docx]

**Supplementary table 1: p values for figure 5a**

| **Th subsets** | **p-value** | **Summary** |
| --- | --- | --- |
| **Th1* (CCR6+CXCR3+CCR4-)** | | |
| ATB at diagnosis (N=12) vs. ATB at mid treatment (N=13) | **0.0064** | ****** |
| ATB at diagnosis (N=12) vs. QFT+ (N=59) | >0.9999 | ns |
| ATB at diagnosis (N=12) vs. QFT- (N=16) | >0.9999 | ns |
| ATB at mid treatment (N=13) vs. QFT+ (N=59) | **0.0013** | ****** |
| ATB at mid treatment (N=13) vs. QFT- (N=16) | **0.0056** | ****** |
| QFT+ (N=59) vs. QFT- (N=16) | >0.9999 | ns |
|  |  |  |
|  |  |  |
| **Th17(CCR6+CXCR3-CCR4+)** | | |
| ATB at diagnosis (N=12) vs. ATB at mid treatment (N=13) | >0.9999 | ns |
| ATB at diagnosis (N=12) vs. QFT+ (N=59) | **0.0002** | ******* |
| ATB at diagnosis (N=12) vs. QFT- (N=16) | **0.0005** | ******* |
| ATB at mid treatment (N=13) vs. QFT+ (N=59) | **0.0082** | ****** |
| ATB at mid treatment (N=13) vs. QFT- (N=16) | **0.0095** | ****** |
| QFT+ (N=59) vs. QFT- (N=16) | >0.9999 | ns |
|  |  |  |
| **CCR6+CXCR3+CCR4+** | | |
| ATB at diagnosis (N=12) vs. ATB at mid treatment (N=13) | **0.0174** | ***** |
| ATB at diagnosis (N=12) vs. QFT+ (N=59) | **0.0063** | ****** |
| ATB at diagnosis (N=12) vs. QFT- (N=16) | 0.4823 | ns |
| ATB at mid treatment (N=13) vs. QFT+ (N=59) | >0.9999 | ns |
| ATB at mid treatment (N=13) vs. QFT- (N=16) | 0.9613 | ns |
| QFT+ (N=59) vs. QFT- (N=16) | >0.9999 | ns |
|  |  |  |
|  |  |  |
| **CCR6+CXCR3-CCR4-** | | |
| ATB at diagnosis (N=12) vs. ATB at mid treatment (N=13) | >0.9999 | ns |
| ATB at diagnosis (N=12) vs. QFT+ (N=59) | 0.1238 | ns |
| ATB at diagnosis (N=12) vs. QFT- (N=16) | 0.1119 | ns |
| ATB at mid treatment (N=13) vs. QFT+ (N=59) | 0.3391 | ns |
| ATB at mid treatment (N=13) vs. QFT- (N=16) | 0.2684 | ns |
| QFT+ (N=59) vs. QFT- (N=16) | >0.9999 | ns |
|  |  |  |
| **Th1 (CCR6-CXCR3+CCR4- )** | | |
| ATB at diagnosis (N=12) vs. ATB at mid treatment (N=13) | >0.9999 | ns |
| ATB at diagnosis (N=12) vs. QFT+ (N=59) | **0.0324** | ***** |
| ATB at diagnosis (N=12) vs. QFT- (N=16) | **0.0054** | ****** |
| ATB at mid treatment (N=13) vs. QFT+ (N=59) | **0.0001** | ******* |
| ATB at mid treatment (N=13) vs. QFT- (N=16) | **<0.0001** | ******** |
| QFT+ (N=59) vs. QFT- (N=16) | >0.9999 | ns |
|  |  |  |
| **Th2 (CCR6-CXCR3-CCR4+ )** | | |
| ATB at diagnosis (N=12) vs. ATB at mid treatment (N=13) | 0.5432 | ns |
| ATB at diagnosis (N=12) vs. QFT+ (N=59) | 0.8471 | ns |
| ATB at diagnosis (N=12) vs. QFT- (N=16) | 0.0628 | ns |
| ATB at mid treatment (N=13) vs. QFT+ (N=59) | **0.0011** | ****** |
| ATB at mid treatment (N=13) vs. QFT- (N=16) | **<0.0001** | ******** |
| QFT+ (N=59) vs. QFT- (N=16) | 0.4165 | ns |
|  |  |  |
| **CCR6-CXCR3+CCR4+** | | |
| ATB at diagnosis (N=12) vs. ATB at mid treatment (N=13) | 0.3973 | ns |
| ATB at diagnosis (N=12) vs. QFT+ (N=59) | >0.9999 | ns |
| ATB at diagnosis (N=12) vs. QFT- (N=16) | >0.9999 | ns |
| ATB at mid treatment (N=13) vs. QFT+ (N=59) | 0.3644 | ns |
| ATB at mid treatment (N=13) vs. QFT- (N=16) | 0.0794 | ns |
| QFT+ (N=59) vs. QFT- (N=16) | >0.9999 | ns |
|  |  |  |
| **CCR6-CXCR3-CCR4-** | | |
| ATB at diagnosis (N=12) vs. ATB at mid treatment (N=13) | 0.2102 | ns |
| ATB at diagnosis (N=12) vs. QFT+ (N=59) | **0.0134** | ***** |
| ATB at diagnosis (N=12) vs. QFT- (N=16) | >0.9999 | ns |
| ATB at mid treatment (N=13) vs. QFT+ (N=59) | >0.9999 | ns |
| ATB at mid treatment (N=13) vs. QFT- (N=16) | >0.9999 | ns |
| QFT+ (N=59) vs. QFT- (N=16) | 0.3912 | ns |
